# Editorial: Recent advances in radiation medical countermeasures

**DOI:** 10.3389/fphar.2022.983702

**Published:** 2022-09-13

**Authors:** Ales Tichy, Dianne Ricobonno, Lynnette H. Cary, Christophe Badie

**Affiliations:** ^1^ Department of Radiobiology, Faculty of Military Health Sciences, University of Defence, Hradec Králové, Czechia; ^2^ Institut de Recherche Biomédicale des Armées (IRBA), Bretigny sur Orge, France; ^3^ Uniformed Services University of the Health Sciences, Bethesda, MD, United States; ^4^ Health Security Agency, Didcot, United Kingdom

**Keywords:** radioprotection, ionizing radiation (IR), growth factors, medical countermeasure, acute radiation syndrome (ARS)

We are presenting a topic research study focused on recent advances in radiation medical countermeasures. Unfortunately, the unstable geo-political situation makes this field very current. The majority of the articles deal with the mitigation of ARS. You can start your reading with a review of radioprotective agents summarizing the repurposing approach of previously approved pharmaceuticals (Singh and Seed).

A couple of original articles are engaged with the application of growth factors in rodent models such as IGF-1 treatment for mitigating GIT damage (Pejchal et al.) and an effective combination of pegylated G-CSF and ghrelin (Kiang et al.). Another group applied a polypharmaceutical approach of pegylated triple combination (hG-SCF, mMG-CSF, and hIL-11), and they even potentiated the effect by combination with lisinopril (Gasperetti et al.). Interestingly, lisinopril was studied by Medhora et al., who proposed that radiation increases its bioavailability by vascular regression.

Other authors showed that survival and hematopoietic recovery can be supported by gamma-tocotrienol (Kumar et al.) and in the case of thoracic irradiation, by hyaluronic nanoparticles (Lierova et al.) or RRx-001 (glucose 6-phosphate dehydrogenase inhibitor; Jurgensen et al.). Additionally, Nanduri et al. evaluated the radioprotective potential of secretory extracellular vesicles as it is seen that a plethora of new radioprotective agents is getting forward to regulatory approval.

Two other overview articles bring interesting radiobiological insights. The review of François et al. discussed the possibility to modulate the inflammatory storm associated with COVID-19 pulmonary infection by exposing patients to ionizing radiation at very low doses. Hollingsworth et al. addressed animal model considerations for designing studies and the potential to use the microbiome as a biomarker to assess radiation exposure and predict the outcome.

Last but not least, we would like to draw your attention to further articles on internal contamination (Griffiths et al.), which compared local and systemic DTPA treatment regimens *in vivo*, and on a novel panel of radiation-responsive biomarkers, which applied an innovative high-throughput proteomics screening approach (Sproull et al.). Finally, Hermann et al. studied the effect of bardoxolone-methyl and successfully radiosensitized oral squamous cancer cells.

As it is seen, our field makes continuous progress forward, and this topic research comprises the latest advances. We hope you will find these articles interesting and the Editorial team wishes you an inspiring tour into the current radiation medical countermeasures.

